# Supplementary material for: Content-rich biological network constructed by mining PubMed abstracts
Source: BMC Bioinformatics. 2004 Oct 8;5:147. doi: 10.1186/1471-2105-5-147 (PMC528731; doi:10.1186/1471-2105-5-147)
Supplement: Additional File 2 — The original results of the above study (non-essential files are deleted to keep the file size under the limit set by BMC bioinformatics). [file 1471-2105-5-147-S2.bz2 › chilibotAdditionalFile2/dip50/684ID9215632E17999/html/CRP_CYTR.html]

 


 **CRP** and **CYTR** 
  
Found 51 abstracts in PubMed, retrieved 50.  
 

 What does Google say? 
 PDF only 
| .edu only 

---

**Interactive relationship** (e.g. stimulation, inhibition, etc)

**Inhibitory relationship**- Moreover, the results provide insight into the mechanism by which  **CytR**  might prevent  **CRP**  mediated transcription.  Ref: 10320573 Mol Microbiol, 1999
- Single amino acid substitutions in the cAMP receptor protein  [ **CRP** ]  specifically abolish regulation by the  **CytR**  repressor in Escherichia coli.  Ref: 1647022 Proc Natl Acad Sci U S A, 1991
- and 2 negative regulation by the  **cytR**  repressor is accomplished by preventing the cAMP  **CRP**  complex from binding to the second target.  Ref: 6329724 EMBO J, 1982
- Furthermore, the reduction in  **CytR**  titration parallels the reduction in binding of cAMP  **CRP**  to the mutated  **CRP**  sites in vitro.  Ref: 1962841 Mol Microbiol, 1990
- The Escherichia coli  **CytR**  regulator belongs to the LacI family of sequence specific DNA binding proteins and prevents  **CRP**  mediated transcription in the  **CytR**  regulon.  Ref: 9466254 Mol Microbiol, 1998
- By introducing  **CRP**  proteins that exhibit an altered DNA binding specificity into a strain containing a mutant deoP2 promoter in which cAMP  **CRP**  activation was decreased and  **CytR**  regulation completely abolished, we show that  **CytR**  regulation of this promoter can be reestablished by restored the DNA binding of the cAMP  **CRP**  complex.  Ref: 1661372 Mol Gen Genet, 1991
- The data obtained using  **crp** \* mutation confirm the proposed model of the udp gene regulation, according to which  **CytR**  repressor protein interferes with  **CRP**  binding site in the promoter operator region of the udp gene and thereby prevents the positive action of cAMP  **CRP**  complex on the udp expression.  Ref: 2668121 Genetika, 1989
**Neutral relationship**- Role of protein protein bridging interactions on cooperative assembly of DNA bound  **CRP**   **CytR**   **CRP**  complex and regulation of the Escherichia coli  **CytR**  regulon.  Ref: 12667072 Biochemistry, 2003
- These results strongly indicate that cooperative DNA binding of  **CytR**  and cAMP  **CRP**  is achieved through protein protein interactions.  Ref: 1647022 Proc Natl Acad Sci U S A, 1991
- DNA induced changes were observed also in the region involved in  **CRP**   **CytR**  interaction and in  **CRP**  intersubunit contact regions.  Ref: 10388556 J Mol Biol, 1999
- no co operativity is observed in the interaction of  **CytR**  with the two  **CRP**  binding sites.  Ref: 1962841 Mol Microbiol, 1990
- Binding to these multiple sites yields both cooperative and competitive interactions between  **CytR**  and  **CRP** .  Ref: 8969182 J Biol Chem, 1996
- The latter is stabilized by cooperative interactions between  **CRP**  and  **CytR** .  Ref: 8969182 J Biol Chem, 1996
- Transcription initiation from  **CytR**  regulated promoters requires cAMP  **CRP** .  Ref: 8596434 Mol Microbiol, 1995
- The cAMP receptor protein  [ **CRP** ]   **CRP**  and the LacI related  **CytR**  antiactivator bind cooperatively to adjacent DNA sites at or near promoters, an interaction that involves direct protein contacts.  Ref: 9215632 Cell, 1997
- In the presence of  **CytR**  the two regulators bind cooperatively to cddP forming a nucleoprotein complex in which cAMP  **CRP**  binds to  **CRP**  1 and  **CRP**  3 and  **CytR**  occupies the sequence between these sites.  Ref: 1327747 EMBO J, 1992
- the formation of an active repression complex requires the combined interactions of cAMP  **CRP**  and  **CytR**  at tsx p2.  Ref: 1715855 J Bacteriol, 1991
- The  **CytR**  repressor and the cAMP receptor protein  [ **CRP** ]   **CRP**  bind cooperatively to several promoters in Escherichia coli to repress transcription initiation.  Ref: 9155036 EMBO J, 1997
- We suggest that the transition from repression to activation involves a switch in the protein protein interactions made by cAMP  **CRP**  from  **CytR**  to RNAP.  Ref: 9086266 J Mol Biol, 1997
- **CytR**  controls the balance between  **CRP**  mediated recruitment and activation of RNA polymerase and transcriptional repression.  Ref: 12667072 Biochemistry, 2003
- In E. coli repression of transcription initiation by the  **CytR**  protein relies on  **CytR**  DNA interactions as well as on interactions between  **CytR**  and the cAMP  **CRP**  activator complex.  Ref: 8451188 Nucleic Acids Res, 1993
- Identification of the subunit of cAMP receptor protein  [ **CRP** ]   **CRP**  that functionally interacts with  **CytR**  in  **CRP**   **CytR**  mediated transcriptional repression.  Ref: 10766824 J Biol Chem, 2000
- These findings indicate that both  **CRP**  targets are required for efficient  **CytR**  repression of deoCp2.  Ref: 2170326 J Bacteriol, 1990
- iii introduction of point mutations in either  **CRP**  target resulted in loss of  **CytR**  regulation.  Ref: 2170326 J Bacteriol, 1990
- When acting from a site close to the DNA target for RNA polymerase,  **CytR**  interacts with  **CRP**  to repress transcription.  Ref: 8816767 Proc Natl Acad Sci U S A, 1996
- Transcription is activated by  **CRP**  and repressed by a multi protein  **CRP** . **CytR** . **CRP**  complex.  Ref: 8969182 J Biol Chem, 1996
- However, cooperativity also means that  **CytR**  can recruit  **CRP**  and, by doing so, can act as a co activator.  Ref: 10347150 J Biol Chem, 1999
- The  **CytR**  repressor protects a DNA segment that is located between the two  **CRP**  sites and partially overlaps the  **CRP**  1 target.  Ref: 1715855 J Bacteriol, 1991
- The three most upstream rot promoters are activated by the global gene regulatory cAMP  **CRP**  complex and negatively regulated by the  **CytR**  repressor protein.  Ref: 7715459 Mol Microbiol, 1994
- Biochemical and genetic studies have shown that  **CytR**  relies on interactions with the cAMP. **CRP**  complex in order to bind promoter DNA and repress transcription.  Ref: 1655726 J Biol Chem, 1991
- Analysis of  **CRP**   **CytR**  interactions at the Escherichia coli udp promoter.  Ref: 8626289 J Bacteriol, 1996
- Initiation of transcription from the cytRP promoter in Escherichia coli is activated by the cAMP  **CRP**  complex and negatively regulated by the  **CytR**  repressor protein.  Ref: 1328649 J Mol Biol, 1992
- Thus, cAMP  **CRP**  induces conformational changes in  **CytR**  so that the repressor fits the natural targets.  Ref: 9155036 EMBO J, 1997
- The central role of cooperativity in regulation is highlighted by the fact that binding of the inducer, cytidine, to  **CytR**  is coupled to  **CytR**   **CRP**  cooperativity.  Ref: 10347150 J Biol Chem, 1999
- Here too, we find that  **CytR**  both cooperates and competes for  **CRP**  binding.  Ref: 10347150 J Biol Chem, 1999
- Restored DNA binding of the cAMP  **CRP**  activator complex reestablishes negative regulation by the  **CytR**  repressor in the deoP2 promoter in Escherichia coli.  Ref: 1661372 Mol Gen Genet, 1991
- The tsx p2 promoter is one of at least seven Escherichia coli promoters that are activated by the cyclic AMP cAMP cAMP receptor protein  [ **CRP** ]   **CRP**  complex and negatively regulated by the  **CytR**  repressor.  Ref: 1715855 J Bacteriol, 1991
- Transcription is repressed by a three protein complex cAMP receptor protein  [ **CRP** ]   **CRP**   **CytR**   **CRP**  that is stabilized by cooperative interactions between  **CRP**  and  **CytR** .  Ref: 10347150 J Biol Chem, 1999
- The repression region is chemically complementary to the characterized region on  **CytR**  that is essential for protein protein communication to  **CRP** .  Ref: 10320573 Mol Microbiol, 1999
- These data indicate that the  **CytR**  repressor relies on the presence of the cAMP  **CRP**  activator complex to regulate tsx p2 promoter activity.  Ref: 1715855 J Bacteriol, 1991
- At promoters of the Escherichia coli  **CytR**  regulon, the cAMP receptor protein  [ **CRP** ]   **CRP**  interacts with the repressor  **CytR**  to form transcriptionally inactive  **CRP**   **CytR**  promoter or  **CRP**  2  **CytR**  promoter complexes.  Ref: 10766824 J Biol Chem, 2000
- These data suggest that  **CytR**  forms a bridge between tandem cAMP  **CRP**  complexes,.  Ref: 1649947 Mol Microbiol, 1991
- In combination, cAMP  **CRP**  and  **CytR**  bind co operatively to cytRP forming a nucleoprotein complex in which the proteins directly interact with each other and bind to the same face of the DNA helix.  Ref: 1328649 J Mol Biol, 1992
- Repression takes place by the formation of a  **CytR**  cAMP  **CRP**  DNA nucleoprotein complex consisting of the two cAMP  **CRP**  molecules and  **CytR**  bound in between.  Ref: 7715459 Mol Microbiol, 1994
- Here, using oriented heterodimer analysis, we show that only one subunit of the  **CRP**  dimer, the subunit proximal to  **CytR** , functionally interacts with  **CytR**  in  **CRP**   **CytR**  promoter and  **CRP**  2  **CytR**  promoter complexes.  Ref: 10766824 J Biol Chem, 2000
- These observations can be reconciled in a competition model for  **CytR**  regulation in which  **CytR**  and RNAP compete for cooperative binding with cAMP  **CRP**  to the promoters in vivo.  Ref: 9086266 J Mol Biol, 1997
- These experiments show that the site of action of  **CytR**  can be specified by protein protein interactions to cAMP  **CRP** .  Ref: 8221894 Cell, 1993
- Transcription from cistrons of the Escherichia coli  **CytR**  regulon is activated by E. coli cAMP receptor protein  [ **CRP** ]   **CRP**  and repressed by a multiprotein complex composed of  **CRP**  and  **CytR** .  Ref: 9202008 J Biol Chem, 1997
- It is shown that cya and  **crp**  mutations completely release the influence of the regulatory gene  **cytR**  on the activity of uridine phosphorylase udp gene and thymidine phosphorylase tpp gene.  Ref: 203511 Genetika, 1978
- In one such promoter, deoP2,  **CytR**  binds to the region between the tandem  **CRP**  binding sites with a relatively low affinity.  Ref: 1647022 Proc Natl Acad Sci U S A, 1991
- Similar extended arrays, but with different arrangements of overlapping  **CytR**  and  **CRP**  sites, are found at the other  **CytR**  regulated promoters.  Ref: 8969182 J Biol Chem, 1996
- Repression by  **CytR**  depends on the formation of nucleoprotein complexes in which  **CytR**  binds cooperatively to the DNA with one or two cAMP  **CRP**  complexes.  Ref: 9086266 J Mol Biol, 1997
- The  **CytR**  repressor protein relies on protein protein interactions to the cAMP  **CRP**  complex to bind its operators with sufficiently high affinity to repress transcription.  Ref: 8764393 J Mol Biol, 1996
- By combining genetic studies and footprinting analyses, we demonstrate that regulated expression of the  **CytR**  controlled cdd promoter requires three  **CRP**  binding sites a high affinity site  **CRP**  1 and two overlapping low affinity sites  **CRP**  2 and  **CRP**  3 centred at positions 4 1 and 93, respectively.  Ref: 1327747 EMBO J, 1992
- The gene encoding the periplasmic cyclophilin homologue, PPIase A, in Escherichia coli, is expressed from four promoters, three of which are activated by the cAMP  **CRP**  complex and negatively regulated by the  **CytR**  repressor.  Ref: 7715459 Mol Microbiol, 1994
- Promoters in Escherichia coli that are negatively regulated by the  **CytR**  repressor are also activated by the cAMP receptor protein  [ **CRP** ]   **CRP**  complexed to cAMP.  Ref: 1655726 J Biol Chem, 1991
- Promoters in Escherichia coli that are negatively regulated by the  **CytR**  repressor are also activated by the cAMP receptor protein  [ **CRP** ]   **CRP**  complexed to cAMP.  Ref: 1647022 Proc Natl Acad Sci U S A, 1991
- Repression by  **CytR**  depends on the formation of nucleoprotein complexes in which the  **CytR**  repressor and the cAMP  **CRP**  activator complex bind co operatively to the DNA.  Ref: 8596434 Mol Microbiol, 1995
- Maximal repression by the  **CytR**  protein depends on the formation of nucleoprotein complexes in which  **CytR**  interacts with DNA and with cAMP cAMP receptor protein  [ **CRP** ] .  Ref: 8221894 Cell, 1993
- These findings support a general model for  **CytR**  binding and  **CytR**  regulation, in which i  **CytR**  and the  **CRP**  cAMP complex bind to similar or identical targets, ii two or more targets are necessary for proper binding of  **CytR**  to a promoter region, and iii  **CytR**  represses transcription by antagonizing cAMP  **CRP**  activation.  Ref: 2575702 Mol Microbiol, 1989
- Here, the quaternary structure of  **CytR**  and the mechanism underlying the cooperative binding of  **CytR**  and cAMP  **CRP**  have been analyzed.  Ref: 8764393 J Mol Biol, 1996
- In addition, the  **CytR**  repressor triggers a DNA repositioning of a cAMP  **CRP**  complex in the 35 region upon binding to its operator.  Ref: 8596434 Mol Microbiol, 1995
- instead we favour a model in which the interaction of  **CytR**  with deoP2 depends on the presence of tandem DNA bound cAMP  **CRP**  complexes.  Ref: 1962841 Mol Microbiol, 1990
- Here, we demonstrate that in order to establish  **CytR**  regulation in a cAMP  **CRP**  dependent class II promoter with a single  **CRP**  site  **CRP**  site centred around position 40.5 in which the  **CytR**  operator is located upstream of the  **CRP**  site, high affinity binding sites for both regulators are required.  Ref: 9086266 J Mol Biol, 1997
- Specifically, in class II promoters with a single  **CRP**  site, the efficiency of  **CytR**  regulation was found to correlate inversely with cAMP  **CRP**  independent promoter activity.  Ref: 9086266 J Mol Biol, 1997
- We have suggested that this variability affects the flexibility of  **CytR**  DNA contacts, thereby affecting the critical protein protein interactions between  **CytR**  and the cAMP receptor protein  [ **CRP** ]   **CRP**  that underlie differential repression and activation of  **CytR**  regulated genes.  Ref: 11866516 J Mol Biol, 2002
- Moreover, mutant cdd promoters in which  **CRP**  2 and  **CRP**  3 have been deleted are partially regulated by  **CytR** , and cAMP  **CRP**  and  **CytR**  still bind cooperatively to these promoters.  Ref: 1327747 EMBO J, 1992
- Tandem DNA bound cAMP  **CRP**  complexes are required for transcriptional repression of the deoP2 promoter by the  **CytR**  repressor in Escherichia coli.  Ref: 1962841 Mol Microbiol, 1990
- In the CRP2  **CytR**  complex all protein DNA phosphate contacts at  **CRP**  1 and  **CRP**  2 are retained, and in addition two new minor groove contacts, ascribed to phosphate  **CytR**  interactions, are observed at 60 between the  **CRP**  sites.  Ref: 8394345 J Biol Chem, 1993
- Finally, the results show that  **CytR**  and RNA polymerase are rivals that compete for binding with  **CRP**  at deoP2.  Ref: 8394345 J Biol Chem, 1993
- ... the  **cytR**  encoded repressor regulates deo operon expression only from the cAMP  **CRP**  dependent promoter operator site, PO2.  Ref: 6098525 Gene, 1984
- In the deoP2 promoter of Escherichia coli, a transcription activator, cAMP  **CRP** , binds at two sites, centered at 41.5 and 93.5 from the start site of transcription, while a repressor,  **CytR** , binds to a space between the two cAMP  **CRP**  complexes.  Ref: 11574471 EMBO J, 2001
- **CytR**  cAMP  **CRP**  nucleoprotein formation in E. coli the  **CytR**  repressor binds its operator as a stable dimer in a ternary complex with cAMP  **CRP** .  Ref: 8764393 J Mol Biol, 1996
- The Escherichia coli cAMP receptor protein  [ **CRP** ]   **CRP**  is essential for the activation and repression of transcription initiation at promoters in the  **CytR**  regulon.  Ref: 10320573 Mol Microbiol, 1999
- These results indicate that  **CytR**  binding to deoP2 relies on both repressor DNA interactions and protein protein interactions to cAMP. **CRP** .  Ref: 1655726 J Biol Chem, 1991
- Hence,  **CytR**  regulation of deoP2 can be modulated by simply varying DNA binding of cAMP  **CRP** .  Ref: 1661372 Mol Gen Genet, 1991
- Rather,  **CytR**  possesses a highly flexible interdomain linker that allows it to form complexes with  **CRP**  at promoters with quite different architecture.  Ref: 9466254 Mol Microbiol, 1998
- **CytR**  binds to a region immediately downstream from, and partially overlapping, the  **CRP**  site, and induces a modest bend into the DNA.  Ref: 1328649 J Mol Biol, 1992
- Analysis of the nupC promoter region revealed the presence of at least two putative  **CRP**  binding sites, centred at 40bp and 89bp, which probably flank a  **CytR**  binding site.  Ref: 8022285 Mol Microbiol, 1994
- Gel retardation and DNase I footprinting analyses show that  **CytR**  is a true sequence specific DNA binding protein that binds to the sequence between the two  **CRP**  sites in deoP2 with a relatively low affinity.  Ref: 1655726 J Biol Chem, 1991
- In this system, the cAMP receptor protein  [ **CRP** ]   **CRP**  assists the binding of RNA polymerase as well as a specific negative regulator,  **CytR** .  Ref: 8816767 Proc Natl Acad Sci U S A, 1996
- Our data confirm and extend the view that  **CytR**  is a highly flexible DNA binder that can adapt many different conformations for co operative binding with  **CRP** .  Ref: 9466254 Mol Microbiol, 1998
- In  **CytR**  regulated promoters in Escherichia coli, the cAMP  **CRP**  complex acts as a transcriptional activator as well as a co repressor for the  **CytR**  protein.  Ref: 9086266 J Mol Biol, 1997
- Based on recent results about  **CRP**  mediated regulation of transcription initiation and the finding that  **CRP**  possesses discrete surface exposed patches for protein protein interaction with RNA polymerase and  **CytR** , a molecular model for this dual regulation is discussed.  Ref: 8816767 Proc Natl Acad Sci U S A, 1996
- Most strikingly,  **CytR**  can adopt widely different conformations that are equally favored energetically for complex formation with cAMP  **CRP** .  Ref: 9155036 EMBO J, 1997
- ii deletion of the uppermost cAMP  **CRP**  target  **CRP**  2 resulted in loss of  **CytR**  regulation, but had only a minor effect on positive control by the cAMP  **CRP**  complex.  Ref: 2170326 J Bacteriol, 1990
- **CRP**  performs these activities by making direct protein protein interactions to the alpha subunits of RNA polymerase and to the  **CytR**  regulator.  Ref: 10320573 Mol Microbiol, 1999
- The  **CytR**  anti activator diverges from the other family members by relying on the cooperative DNA binding with the global regulator  **CRP** .  Ref: 10329134 J Mol Biol, 1999
- Here we show that  **CytR**  binding to the deoP2 promoter relies on interaction with the master gene regulatory protein,  **CRP** , and, furthermore, that cAMP  **CRP**  and  **CytR**  bind co operatively to deoP2.  Ref: 1649947 Mol Microbiol, 1991
- Cooperative interactions between  **CytR** , when bound to an operator CytO located upstream of a  **CytR**  regulated promoter, and  **CRP** , when bound to flanking tandem promoters, are critical to the regulatory role of  **CytR** .  Ref: 12667072 Biochemistry, 2003
- Transcription initiation at  **CytR**  regulated promoters in Escherichia coli is controlled by a combinatorial regulatory system in which the cAMP receptor protein  [ **CRP** ]   **CRP**  functions as both an activator and a co repressor.  Ref: 1327747 EMBO J, 1992
- Recently, we showed that  **CytR**  binds specifically to multiple sites in the E. coli deoP promoter, thereby providing competition for  **CRP**  binding to  **CRP**  operator site 1 CRP1 and CRP2 as well as cooperativity.  Ref: 10347150 J Biol Chem, 1999
- Using a modified Ferguson analysis in which protein DNA complexes are separated in a non denaturing gel system, we show that  **CytR**  binds its operators as a dimer alone as well as in a ternary complex with cAMP  **CRP** .  Ref: 8764393 J Mol Biol, 1996
- The unlinked operons that comprise the Escherichia coli  **CytR**  regulon are controlled coordinately through interactions between two gene regulatory proteins, the cAMP receptor protein  [ **CRP** ]   **CRP**  and the cytidine repressor  **CytR** .  Ref: 12667072 Biochemistry, 2003
- Furthermore,  **CytR**  proteins deleted for the DNA binding domain repress deoP2 in vivo and interact with deoP2 in vitro in a strictly cAMP  **CRP**  dependent fashion.  Ref: 8221894 Cell, 1993
- The cAMP  **CRP**  complex and the  **CytR**  anti activator bind co operatively to the promoter region forming a repression complex that overlaps the sigmaE dependent P3 promoter and the sigma70 dependent P4 and P5 promoters.  Ref: 9767576 Mol Microbiol, 1998
- Unlike the other members of this protein family,  **CytR**  binds with only modest affinity to its operators and transcription repression thus relies on the formation of nucleoprotein complexes with the cAMP  **CRP**  complex.  Ref: 9466254 Mol Microbiol, 1998
- On this ground it is assumed that the  **cytR**  gene product acts at the level of promotors of the corresponding structural genes, causing their insensitivity to the positive action of cAMP  **CRP**  complex.  Ref: 203511 Genetika, 1978
- The  **CytR**  protein was shown to act as a transcription repressor or activator, depending on the configuration of the promoter and on the relative location and quantity of binding sites for  **CytR**  and  **CRP**  proteins.  Ref: 9777353 Genetika, 1998
- Also, point mutations in either  **CRP**  site reduce or abolish  **CytR**  titration.  Ref: 1962841 Mol Microbiol, 1990
- The stimulation of udp expression by  **CytR**  in certain mutants may reflect its stabilization of cAMP  **CRP**  binding to target DNA and illustrates that only modest evolutionary changes could allow particular multiprotein complexes to serve as either repressors or transcriptional activators.  Ref: 8626289 J Bacteriol, 1996
- and iv regulation by  **CytR**  of deletion mutants lacking  **CRP**  2 could be specifically reestablished by increasing the intracellular concentration of  **CytR** .  Ref: 2170326 J Bacteriol, 1990

**Non-interactive relationship** (e.g. studied together, co-existance, homology, etc.)

- Expression of the deo operon of Escherichia coli is subjected to double negative control by DeoR and  **CytR**  repressors and to the positive control by cAMP  **CRP**  complex.  Ref: 2191896 Genetika, 1990
- In the absence of  **CytR** , cAMP  **CRP**  interacts at one set of sites  **CRP**  1 and  **CRP**  2 and both of these binding sites are required for full promoter activation.  Ref: 1327747 EMBO J, 1992
- Here, we identify a collection of amino acid substitutions in  **CytR**  that reestablish protein protein communication to mutant  **CRP**  proteins specifically defective in cooperative binding with wild type  **CytR** .  Ref: 9215632 Cell, 1997
- The same data concerning the effect of cya and  **crp**  mutations on  **cytR**  regulation have been reported 8, but these authors favoured the hypothesis that the  **cytR**  gene product is a repressor protein, which binds to the specific operator.  Ref: 203511 Genetika, 1978
- This study indicates that the minimal DNA region required to obtain  **CytR**  regulation consists of a single binding site for each of cAMP  **CRP**  and  **CytR** .  Ref: 1328649 J Mol Biol, 1992
- Our results established that residues 1 0 08 and 110 are essential for the interaction with  **CytR**  and confirmed that activating region 2 of  **CRP**  is made up of residues 1 1 and 101.  Ref: 10320573 Mol Microbiol, 1999
- Formation of the cAMP  **CRP**   **CytR**  DNA nucleoprotein complex in vitro was perturbed in each mutant.  Ref: 1715855 J Bacteriol, 1991
- Identification of the nucleotide sequence recognized by the cAMP  **CRP**  dependent  **CytR**  repressor protein in the deoP2 promoter in E. coli.  Ref: 8451188 Nucleic Acids Res, 1993
- We have investigated in vivo the coupling between  **CytR**  regulation of the deoP2 promoter in Escherichia coli and the DNA binding specificity of the cAMP  **CRP**  cAMP receptor protein  [ **CRP** ]  complex in order to obtain a more detailed picture of the role played by cAMP  **CRP**  in  **CytR**  regulation.  Ref: 1661372 Mol Gen Genet, 1991
- The mechanisms for the cAMP  **CRP**  mediated transcription activation and  **CytR**  mediated transcription repression were investigated in vitro using purified components.  Ref: 11574471 EMBO J, 2001
- These data indicate that regulation of the udp gene which is controlled by the  **cytR**  repressor protein and by cyclic AMP  **CRP**  complex, is disturbed in the presence of the mutated rho factor.  Ref: 7049835 Genetika, 1982
- These findings show that the bacterium uses positive and negative regulatory modules to differentially control the expression of  **CytR**  and cAMP  **CRP**  regulated genes.  Ref: 8596434 Mol Microbiol, 1995
- Dual function regulators the cAMP receptor protein  [ **CRP** ]  and the  **CytR**  regulator can act either to repress or to activate transcription depending on the context.  Ref: 8816767 Proc Natl Acad Sci U S A, 1996
- In mutants with an improved 10 region, a partial relief from the control of the cAMP  **CRP**  transcription activation complex occurred, and the negative  **CytR**  repressor regulation was reduced.  Ref: 12391883 Genetika, 2002
- Our results support the model in which repression normally involves the binding of  **CytR**  to cAMP  **CRP**  to form a complex which binds to specific sites in the udp promoter, without direct interaction between  **CytR**  protein and a specific operator DNA sequence, and in which induction by specific inducer cytidine involves dissociation of  **CytR**  from cAMP  **CRP**  and the RNA polymerase interaction with cAMP  **CRP**  bound to a site upstream of then transcription start point.  Ref: 8626289 J Bacteriol, 1996
- Binding of cAMP receptor protein  [ **CRP** ]   **CRP**  and  **CytR**  mediates both positive and negative control of transcription from Escherichia coli deoP2.  Ref: 8969182 J Biol Chem, 1996
- Here we have investigated this cooperativity by isolating mutants of the  **CRP**  protein that abolish  **CytR**  regulation without exhibiting a concomitant loss in their ability to activate transcription.  Ref: 1647022 Proc Natl Acad Sci U S A, 1991
- On the basis of the regulatory features of the promoters analysed here, we speculate about the advantages offered by the structural complexity of natural  **CytR**  cAMP  **CRP**  regulated promoters.  Ref: 9086266 J Mol Biol, 1997
- Here we show that the Escherichia coli nupG gene contains one  **CytR**  and four  **CRP**  binding sites in the control region.  Ref: 8596434 Mol Microbiol, 1995
- We have investigated the regulation of the Escherichia coli deoCp2 promoter by the  **CytR**  repressor and the cyclic AMP cAMP receptor protein  [ **CRP** ]   **CRP**  complexed to cAMP.  Ref: 2170326 J Bacteriol, 1990
- cAMP  **CRP**  functions as an adaptor for  **CytR** .  Ref: 1649947 Mol Microbiol, 1991
- Characterization of promoter recognition complexes formed by  **CRP**  and  **CytR**  for repression and by  **CRP**  and RNA polymerase for activation of transcription on the Escherichia coli deoP2 promoter.  Ref: 8394345 J Biol Chem, 1993
- To investigate the chemical and structural basis for these effects, we investigated how cytidine binding affects association between  **CytR**  and  **CRP**  in solution and how it affects the binding of  **CytR**  deletion mutants lacking the DNA binding HTH domain, with tandem  **CRP**  dimers bound to either udpP or deoP2.  Ref: 12667072 Biochemistry, 2003
- This approach enables us to specify the patch on  **CytR**  s surface that contacts  **CRP** .  Ref: 9215632 Cell, 1997
- Transcriptional regulation of the deoP2 promoter by the cyclic AMP cyclic AMP receptor protein complex cAMP  **CRP**  and the  **CytR**  repressor requires two high affinity  **CRP**  targets located around 41 and 93 bp preceding the start site for transcription.  Ref: 2575702 Mol Microbiol, 1989
- Transcription of rpoH, encoding the Escherichia coli heat shock regulator sigma32, is negatively controlled by the cAMP  **CRP**   **CytR**  nucleoprotein complex.  Ref: 9767576 Mol Microbiol, 1998
- A flexible partnership the  **CytR**  anti activator and the cAMP  **CRP**  activator protein, comrades in transcription control.  Ref: 8736525 Mol Microbiol, 1996
- These findings demonstrate that direct coupling between cytidine binding to  **CytR**  and  **CytR**   **CRP**  association does not account for promoter specific effects on cooperativity.  Ref: 12667072 Biochemistry, 2003
- Sequences similar to the sigma 70 promoters P1, P4 and P5, to the sigma E promoter P3 and to boxes DnaA1, DnaA2, cAMP receptor protein  [ **CRP** ]   **CRP**  boxes CRP1, CRP2 and box  **CytR**  present in Escherichia coli K12, were identified in sequences of closely related bacteria such as E.coli, Shigella flexneri, Salmonella enterica serovar Typhimurium, Citrobacter freundii, Enterobacter cloacae and Klebsiella pneumoniae.  Ref: 11139607 Nucleic Acids Res, 2001
- Indeed, taken by itself, the  **crp**  a mutation did not lead to any increase in the expression of udpP lac fusion under the conditions of cAMP limitation on glucose grown cells, in spite of whether or not the  **CytR**  repressor was present.  Ref: 2668121 Genetika, 1989
- Cooperativity with  **CRP**  at CRP1 is nearly eliminated, but the effect on  **CytR**  CRP2 cooperativity is negligible.  Ref: 10347150 J Biol Chem, 1999
- Thus, these data rule out the possibility that the cooperative DNA binding of  **CytR**  and cAMP  **CRP**  involves induced dimerization of  **CytR** , and they suggest that cytidine interrupts the cooperative binding of  **CytR**  and cAMP  **CRP**  solely by perturbing the protein protein interactions between the two proteins.  Ref: 8764393 J Mol Biol, 1996
- Interaction of negative  **CytR**  and positive cAMP  **CRP**  control in the promoter region of the uridine phosphorylase udp gene of Escherichia coli has been studied by using udp lac operon fusions in which the structural lacZ gene is expressed from the wild type promoter udpP or from mutant promoters udpP1 and udpP18.  Ref: 2668121 Genetika, 1989
- **CRP**  cAMP and  **CytR**  regulated promoters in Escherichia coli K12 the cdd promoter.  Ref: 2575702 Mol Microbiol, 1989
- Furthermore, the inducer cytidine does not affect independent DNA binding of  **CytR** , rather the  **CytR**  cAMP. **CRP**  cooperativity is perturbed.  Ref: 1655726 J Biol Chem, 1991
- After introduction of mutations affecting binding sites for  **CRP** , the negative effect of the  **CytR**  protein on promoter transcription was fully abolished.  Ref: 12391883 Genetika, 2002
- However, when co binding with cAMP  **CRP** ,  **CytR**  instead recognizes inverted repeats separated by 10 13 bp, or direct repeats separated by 1 bp.  Ref: 9155036 EMBO J, 1997
- The specific activity of beta galactosidase was examined in these fusions in  **cytR**  and  **cytR**  backgrounds after introduction of specific mutations in  **crp**  locus,  **crp** \* and  **crp**  a altering interaction of  **CRP**  protein with catabolite sensitive promoters.  Ref: 2668121 Genetika, 1989
- In this model, two mutually exclusive ternary complexes can be formed a  **CytR**  cAMP  **CRP**  promoter repression complex and an RNAP cAMP  **CRP**  promoter activation complex.  Ref: 9086266 J Mol Biol, 1997
- Despite only weak association between  **CytR**  and  **CRP**  in solution, our results demonstrate the formation of a relatively stable complex in which the Core domain forms a protein bridge between tandem  **CRP**  dimers when bound to either udpP or deoP2.  Ref: 12667072 Biochemistry, 2003
- To understand the combinatorial control mechanism at deoP2, we have used quantitative footprint and gel shift analysis of  **CRP**  and  **CytR**  binding to evaluate the distribution of ligation states.  Ref: 8969182 J Biol Chem, 1996
- Thus, cytidine interferes with the cooperative DNA binding of cAMP  **CRP**  and  **CytR**  to tsx p2.  Ref: 1715855 J Bacteriol, 1991
- Here we show that i  **CytR**  selectively regulated cAMP  **CRP**  dependent initiations, although transcription started from the same site in deoCp2 in the absence or presence of cAMP  **CRP** .  Ref: 2170326 J Bacteriol, 1990
- However, this cytidine mediated effect varies among promoters, suggesting that coupling between cytidine binding to  **CytR**  and  **CytR**   **CRP**  association is sensitive to promoter structure.  Ref: 12667072 Biochemistry, 2003
- These data confirm the crucial role played by the cAMP  **CRP**  activator complex in  **CytR**  regulation of the deoP2 promoter.  Ref: 1661372 Mol Gen Genet, 1991
- As is characteristic for  **CytR**  regulated promoters, deoP2 encodes tandem binding sites for the activating complex cAMP  **CRP** .  Ref: 1962841 Mol Microbiol, 1990
- We discuss these novel concepts in the context of the  **CRP**   **CytR**  regulatory system, as well as the structural and functional implications for multiprotein DNA complex formation in general.  Ref: 9155036 EMBO J, 1997
- Here, we have used quantitative footprint and mobility shift analysis to investigate  **CRP**  and  **CytR**  binding to the E. coli udp promoter.  Ref: 10347150 J Biol Chem, 1999
- Furthermore, our results permit the construction of a three dimensional structure of the higher order nucleoprotein complex formed by  **CytR**  and  **CRP** .  Ref: 9215632 Cell, 1997
- Protein induced fit the  **CRP**  activator protein changes sequence specific DNA recognition by the  **CytR**  repressor, a highly flexible LacI member.  Ref: 9155036 EMBO J, 1997
- These findings provide clues to an understanding of how cAMP  **CRP**  and  **CytR**  interact at a structurally diverse set of promoters.  Ref: 1327747 EMBO J, 1992
- A novel function of the cAMP  **CRP**  complex in Escherichia coli cAMP  **CRP**  functions as an adaptor for the  **CytR**  repressor in the deo operon.  Ref: 1649947 Mol Microbiol, 1991
- However, sensitivity of the deo operon to catabolite repression is only revealed in bacteria with disrupted synthesis of the  **CytR**  protein, since the function of the latter is to prevent  **CRP**  activation of the deo operon transcription.  Ref: 2191896 Genetika, 1990
- Here, in vitro selection experiments have been used to examine the DNA binding characteristics of  **CytR** , by itself and when co binding with cAMP  **CRP** .  Ref: 9155036 EMBO J, 1997
- The cAMP  **CRP**   **CytR**  nucleoprotein complex in Escherichia coli two pairs of closely linked binding sites for the cAMP  **CRP**  activator complex are involved in combinatorial regulation of the cdd promoter.  Ref: 1327747 EMBO J, 1992
- The effect of the competition at this promoter is to negate the role of  **CytR**  in recruiting  **CRP** .  Ref: 10347150 J Biol Chem, 1999
- We propose that competition and cooperativity in  **CytR**  and  **CRP**  binding are important to differential regulation of these promoters.  Ref: 8969182 J Biol Chem, 1996
- The structure of the cAMP  **CRP**   **CytR**  repression complex and the cAMP  **CRP**  RNA polymerase initiation complex at the deoP2 promoter of E. coli have been probed by DNase I and uranyl footprinting.  Ref: 8394345 J Biol Chem, 1993
- The  **CytR**  repressor both contacts DNA and the cAMP receptor protein  [ **CRP** ]  when binding to the deoP2 promoter.  Ref: 1655726 J Biol Chem, 1991
- The inducer for the  **CytR**  repressor, cytidine, does not prevent in vitro DNA binding of  **CytR** , but releases the repressor from the nucleoprotein complex and leaves the cAMP  **CRP**  activator bound to its two DNA targets.  Ref: 1715855 J Bacteriol, 1991
- Additional data in favor of this model were obtained using  **crp**  a mutation which most probably alters the structure of  **CRP**  protein in such a way that it exhibits more high affinity to the udp promoter, as compared to the  **CytR**  repressor protein.  Ref: 2668121 Genetika, 1989
- In particular, the transcription of genes needed for utilization of nucleosides in Escherichia coli is regulated by a repressor protein,  **CytR** , in concert with the cyclic AMP cAMP activated form of cAMP receptor protein  [ **CRP** ]   **CRP** .  Ref: 8626289 J Bacteriol, 1996
- Gene regulatory modules in Escherichia coli nucleoprotein complexes formed by cAMP  **CRP**  and  **CytR**  at the nupG promoter.  Ref: 8596434 Mol Microbiol, 1995
- On the other hand, the  **crp**  a mutation causes only slight alteration in the expression of udpP18 lac fusion, providing additional evidence that udpP18 mutation seems to comprise a modification of the promoter operator region, where binding sites for  **CRP**  and  **CytR**  proteins overlap.  Ref: 2668121 Genetika, 1989
- Regulation of its expression was studied by using tsx lacZ protein and operon fusion strains carrying mutations in deoR,  **cytR** , and  **crp** .  Ref: 2826388 J Bacteriol, 1988
- Here we report the structure of cddP, another  **CRP**   **CytR**  regulated promoter.  Ref: 2575702 Mol Microbiol, 1989
- Here we show that, depending on promoter architecture, the  **CRP**   **CytR**  nucleoprotein complex has opposite effects on transcription.  Ref: 8816767 Proc Natl Acad Sci U S A, 1996
- **CytR**  binding concomitantly antagonizes the cAMP  **CRP**  induced bend.  Ref: 1328649 J Mol Biol, 1992
- In Process Citation Site directed mutagenesis was conducted in the regulatory region of the Escherichia coli udp gene at promoter sites responsible for binding regulatory proteins  **CRP**  and  **CytR**  as well as RNA polymerase.  Ref: 12391883 Genetika, 2002
- On the other hand, the measurements of uridine phosphorylase activity in the  **cytR**  and cya background indicate that expression of the cloned udp gene escapes partially negative control of the  **CytR**  repressor and positive control of cAMP  **CRP**  complex.  Ref: 2515986 Genetika, 1989
- Here we demonstrate, by means of in vivo titration, that  **CytR**  interaction with deoP2 depends not only on  **CRP**  2, but also on  **CRP**  1 and the length and possibly the sequence separating these two sites.  Ref: 1962841 Mol Microbiol, 1990
- Design of  **CytR**  regulated, cAMP  **CRP**  dependent class II promoters in Escherichia coli RNA polymerase promoter interactions modulate the efficiency of  **CytR**  repression.  Ref: 9086266 J Mol Biol, 1997
- The data presented suggest that the expression of the P2 promoter, unlike that of P1, is not subjected to regulation with participation of the  **CytR**  protein and the cAMP  **CRP**  complex.  Ref: 12068544 Genetika, 2002
- Our results provide information about the architecture of  **CRP**   **CytR**  promoter and  **CRP**  2  **CytR**  promoter complexes and rule out the proposal that masking of activating region 2 of  **CRP**  is responsible for the transcriptional inactivity of the complexes.  Ref: 10766824 J Biol Chem, 2000
- In the presence of the cAMP. **CRP**  complex the two protein species bind cooperatively to deoP2, forming a complex in which  **CytR**  occupies the sequence between the two DNA bound cAMP. **CRP**  complexes.  Ref: 1655726 J Biol Chem, 1991
- Introduction of the rho15 mutation into the udpP1 and udpP18 promoter mutants which are characterized by  **cytR**  and or  **CRP**  independent expression of the udp gene, leads to 2 fold reduction in uridine phosphorylase activity.  Ref: 7049835 Genetika, 1982
- Multiple specific  **CytR**  binding sites at the Escherichia coli deoP2 promoter mediate both cooperative and competitive interactions between  **CytR**  and cAMP receptor protein  [ **CRP** ] .  Ref: 8969182 J Biol Chem, 1996
- Protein protein communication structural model of the repression complex formed by  **CytR**  and the global regulator  **CRP** .  Ref: 9215632 Cell, 1997
- The mechanism of repression by  **CytR**  proposed in this study is that the cAMP  **CRP**  bound at 41.5 undergoes an allosteric change upon direct interaction with  **CytR**  such that it no longer maintains a productive interaction with the N terminal domain of alpha, but instead acts as a repressor to interfere with RNA polymerase acting on deoP2.  Ref: 11574471 EMBO J, 2001
- In combination, the cAMP  **CRP**  and  **CytR**  proteins bind cooperatively to tsx p2, and the nucleoprotein complex formed covers a region of 78 bp extending from the  **CRP**  2 site close to the 10 region.  Ref: 1715855 J Bacteriol, 1991
- Multiple binding sites for the  **CytR**  and  **CRP**  proteins were identified outside the canonical udp gene promoter.  Ref: 9777353 Genetika, 1998
- These data are in accordance with suggestion that udpP1 is a mutation in the site of the promoter operator region that responds to the  **cytR**  gene product, while the corresponding binding site for  **CRP**  protein is still unaltered in this mutant.  Ref: 2668121 Genetika, 1989
- cAMP  **CRP**  activator complex and the  **CytR**  repressor protein bind co operatively to the cytRP promoter in Escherichia coli and  **CytR**  antagonizes the cAMP  **CRP**  induced DNA bend.  Ref: 1328649 J Mol Biol, 1992
